# Randomized Controlled Trial of Perioperative Telemonitoring of Patient Generated Health Data in Gastrointestinal Oncologic Surgery: Assessing Overall Feasibility and Acceptability

**DOI:** 10.1007/s00268-023-07179-y

**Published:** 2023-09-20

**Authors:** Kristen Limbach, Patricia Esslin, Virginia Sun, Darrell Fan, Andreas M. Kaiser, I. Benjamin Paz, Mustafa Raoof, Aaron Lewis, Kurt A. Melstrom, Lily Lai, Yanghee Woo, Gagandeep Singh, Yuman Fong, Laleh G. Melstrom

**Affiliations:** 1 Department of Surgery, City of Hope National Medical Center, 1500 E. Duarte Rd, Duarte, CA 91010 USA; 2 Department of Nursing Research, City of Hope National Medical Center, Duarte, CA USA

## Introduction

Since the beginning of the COVID-19 pandemic, the use of remote telemonitoring and telehealth platforms has been of steadily increasing interest, and implementation continues to rise [1]. Although this technology has the potential to be applied in a wide spectrum of medical conditions and care environments [1–4], one area in which remote telemonitoring may have a significant impact on both safety and value is in the perioperative period [5]. Studies of telemonitoring in this context have described the use of patient-generated health data (PGHD), particularly after discharge, including the use of activity trackers/step counters as well as electronic patient-reported outcomes (ePROs) [6]. Results have so far been encouraging. For example, a 2021 meta-analysis examining the use of mobile health technology for remote telemonitoring after surgery found that implementation of such monitoring technology was both feasible and associated with significantly fewer emergency department visits and readmissions [5]. However, the exact populations who may derive the greatest benefit have yet to be fully elucidated.

One population that has been identified as potentially deriving significant benefit from remote telemonitoring is patients with cancer [7]. It has been noted that complications of cancer treatment, including surgery, vary widely with resultant variation in associated costs [8]. Thus, reduction in complication and readmission rates represents a possible source of great value in healthcare delivery, and remote telemonitoring technology may be a potential tool for contributing to such value. However, a paucity of data exist regarding the use of this technology in patients undergoing complex cancer surgery. One randomized controlled trial in patients undergoing liver transplant has been published by Lee and colleagues, and this reported that the intervention was both feasible and associated with a decreased 90-day readmission rate as well as an improved QoL [9]. However, significant differences exist between the liver transplant population and patients undergoing oncologic resection, and there are many challenges in introducing digital technology into the perioperative care of complex cancer patients. These may include digital literacy, access to appropriate electronic equipment, and compliance. Therefore, assessment of feasibility remains a critical first step to the evaluation of benefit for telemonitoring technology in this population.

Thus, the primary aim of this prospective randomized trial is to assess the feasibility and acceptability of perioperative telemonitoring in patients undergoing complex gastrointestinal oncologic surgery. Herein we present preliminary data regarding patient adherence and satisfaction.

## Materials and methods

### Patients

Patients undergoing gastrointestinal oncologic surgery (all colorectal surgery, pancreatectomy, hepatectomy and gastrectomy) at a single institution over 18 years of age who could read and write in English were eligible for inclusion. All eligible patients were screened. Only those patients who had completed all participation by August 31, 2022, were included in the present analysis. All individual patients included in the study provided written informed consent. The study protocol was approved by the institutional review board and was performed in accordance with the ethical standards as laid down by the Declaration of Helsinki.

### Randomization and procedures

**Fig. 1:**
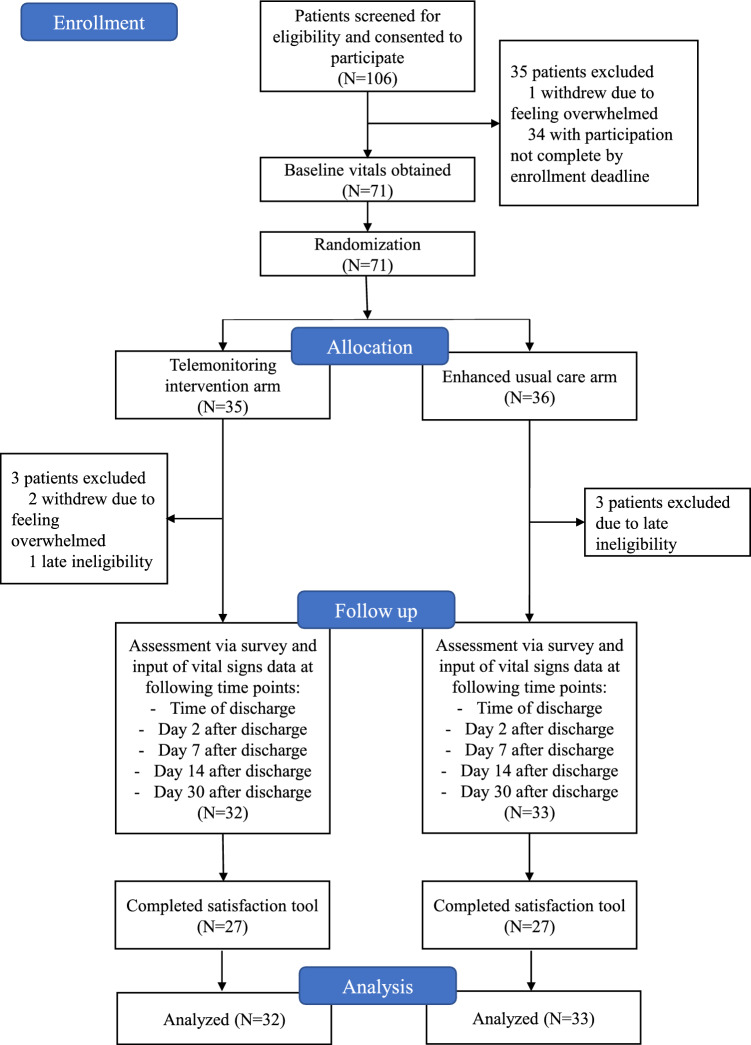
CONSORT diagram

**Fig. 2:**
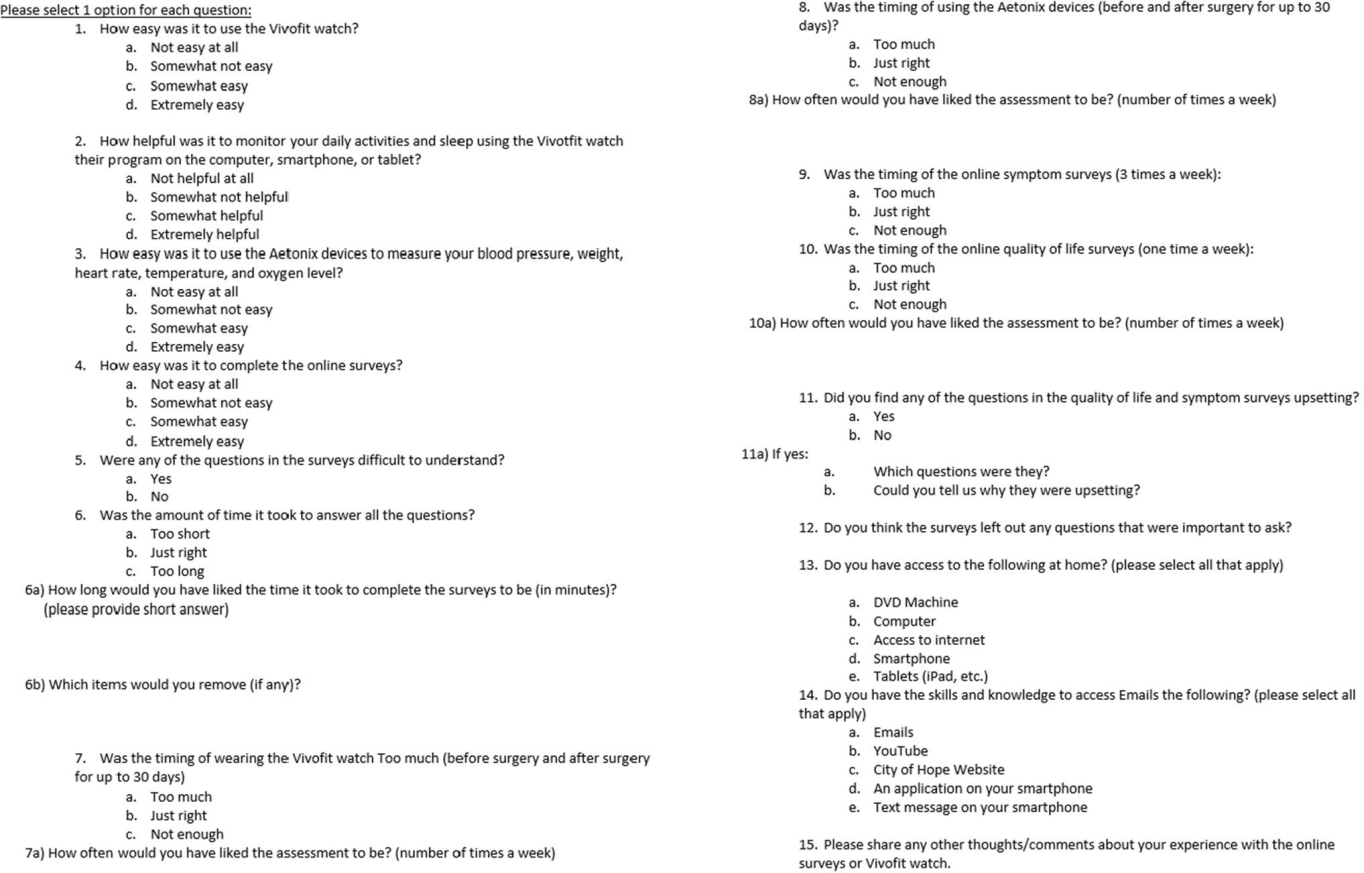
Satisfaction tool

A study algorithm detailing randomization, subsequent allocation, and follow-up is shown in the CONSORT diagram in Fig. 1. Patients meeting inclusion criteria were recruited at a preoperative clinic visit prior to operation by study staff. Patients consenting to participate were preoperatively randomized 1:1 to either the telemonitoring intervention arm or the control arm, designated as enhanced usual care, after baseline measurements of vital signs and weight. Patients in both arms of the study were then provided with a VivoFit activity tracker, Bluetooth-enabled equipment to measure blood pressure, oxygen saturation, heart rate, temperature and weight, and a telehealth monitoring app, aTouchAway by Aetonix (Ottawa, Ontario). The telemonitoring app was uploaded by research staff on the patient’s personal device, and education was provided to each patient on its use. Patients then completed electronic surveys of symptoms and recorded vital signs via the app at specified time points after discharge (time of discharge, day 2 after discharge, day 7, day 14, and day 30) between October 21, 2021, and August 31, 2022. Symptom severity and impact was assessed using two validated instruments: the MD Anderson Symptom Inventory (MDASI) [10] and the EuroQol five-dimensional descriptive system (EQ-5D-5L) [11, 12]. Those in the intervention arm received nursing triage support via telephone outreach when data deviated from predetermined thresholds, while the standard of care triage line was provided to those in the control arm. Thresholds for nursing triage intervention were an increase or decrease in weight of 2 kg, temperature above 38 °C, heart rate greater than 110 beats per minute, systolic blood pressure below 90 mmHg or above 180 mmHg, oxygen saturation less than 90% on room air, and fewer than 1500 steps per day. Patients were not given an explicit step goal. The steps data were automatically synced to data collection. The research nurse checked steps data daily. Additionally, both arms received telephone reminders from research staff when electronic surveys and vital sign input had not been entered at the designated time points. During the study, the control arm was instructed to call the triage line automatically when input values were beyond threshold for vitals and ePROs. The intervention arm is the arm that received calls from the research nurse when there was aberrant data from preset thresholds in the intervention arm. All participating patients were then contacted via telephone after the 30-day time point by study staff to complete a multiple-choice satisfaction tool, as shown in Fig. 2. Vital sign, survey, and satisfaction tool data from each time point were recorded for later analysis. Patients for whom the qualifying operation was canceled or aborted due to spread of disease were considered to have late ineligibility. Equipment was purchased from Garmin and mTelehealth (Orlando, FL). A full-time effort nurse was tasked with enrolling in the study and data monitoring.

### Outcomes

The primary outcome was feasibility as defined by the percent of patients completing electronic surveys at 70% of all time points. Acceptability was assessed by a patient satisfaction tool assessing ease of use, time burden of participation, and technological access after completion of the 30-day tracking period (Fig. 2).

### Statistical analysis

Univariate analysis was performed using SPSS version 28.0 (Armonk, New York). Patient characteristics including demographics and surgical treatment were compared between groups using Student’s t-test for continuous variables, the Pearson Chi-square test for categorical variables, and the Mann–Whitney U test for ordinal variables. The level of significance was set at *p* = 0.05.

## Results

One hundred and six patients were screened, of which 83 (78.3%) consented to participate and 65 (78.3%) completed participation by August 31, 2022 (Fig. 1). Median age was 52 years (range 28–72), and 21 patients (32.3%) were female (Table 1). There were no significant differences in age, sex, or racial demographics between the intervention and control groups. The majority (90.8%) of patients lived with at least one family member, most frequently their spouse. 93.5% completed a high school education or higher. There were no significant differences in living arrangements, education completed, or ASA score between groups. Operations performed were most frequently complex colorectal (15 patients, 23%), liver (29 patients, 44.6%), or combination colorectal and liver procedures (7 patients, 10.7%), with no significant differences in resection site between the two arms of the study (p = 0.245) (Table 2). Both open and minimally invasive surgical operations were performed, with 55.4% of operations classified as open and 44.6% classified as minimally invasive. The proportion of open versus minimally invasive procedures was not significantly different between study groups (*p* = 0.156). The overall attrition rate was 4.6% [3 patients], and four patients had late ineligibility (6.1%).

**Table 1:** Patient demographics

Variable	Overall (N = 65 participants)	Enhanced usual care (N = 33 participants)	Telemonitoring (N = 32 participants)	*P* value
Age, median (range), y	52 (28–78)	51 (28–71)	52.5 (37–78)	0.276
*Sex, N (%)*				0.378
Female	21 (32.3)	9 (27.2)	12 (27.5)	
Male	44 (67.6)	24 (72.7)	20 (62.5)	
*Race, N (%)*				0.353
White	36 (55.3)	19 (57.6)	17 (53.1)	
Black or African-American	3 (4.6)	2 (6)	1 (3.1)	
Asian	12 (18.4)	6 (18.2)	6 (18.8)	
More than one race	4 (6.1)	0 (0)	4 (12.5)	
Other	9 (13.8)	5 (15.10	4 (12.5)	
Decline to state	1 (1.5)	1 (3)	0 (0)	
*Ethnicity, N (%)*				0.321
Hispanic or Latino	20 (30.7)	12 (36.3)	8 (25)	
Non-Hispanic	45 (69.2)	21 (63.6)	24 (75)	
*Living situation, N (%)*				
Spouse	50 (76.9)	25 (75.7)	25 (78.1)	0.821
Children	32 (49.2)	18 (54.5)	14 (43.8)	0.384
Parents/other relatives	7 (10.7)	6 (18.2)	6 (18.8)	0.721
Friends/significant Other	1 (1.5)	1 (3)	0 (0)	0.321
Live Alone	6 (9.2)	2 (6)	4 (12.5)	0.370
Education, N (%)	4 (6.1)	3 (9.1)	1 (3.1)	
Did not complete high school	10 (15.3)	4 (12.1)	6 (18.8)	
Completed high school / GED	15 (23)	8 (24.2)	7 (21.9)	
Some college	20 (30.7)	12 (36.4)	8 (25)	
Graduated college	2 (3)	1 (3)	1 (3.1)	
Some graduate school	14 (21.5)	5 (15.2)	9 (28.1)	
*Completed graduate school*				0.590
ASA, N (%)	1 (1.5)	0 (0)	1 (3.1)	
II	56 (86.1)	29 (87.9)	27 (81.8)	
III	8 (12.3)	4 (12.1)	4 (12.5)	
IV

**Table 2:** Perioperative characteristics

Variable	Overall (N = 65 participants)	Enhanced usual care (N = 33 participants)	Telemonitoring (N = 32 participants)	P value
*Resection site, N (%)*				0.245
Colorectal	15 (23)	11 (33.3)	4 (12.5)	
Liver	29 (44.6)	15 (45.5)	14 (43.8)	
Pancreas	3 (4.6)	1 (3)	2 (6.3)	
Colorectal + Liver	7 (10.7)	2 (6)	5 (15.6)	
Colorectal + GU/GYN/intestinal	5 (7.8)	3 (9.1)	2 (6.3)	
Intestinal	2 (3)	0 (0)	2 (6.3)	
Foregut	2 (3)	0 (0)	2 (6.3)	
Liver + Pancreas/intestinal	2 (3)	1 (3)	1 (3.1)	
*Surgical technique, N (%)*				0.156
Open	36 (55.4)	16 (48.4)	20 (62.5)	
Laparoscopic or MIS/robotic assisted	29 (44.6)	17 (51.5)	12 (37.5)	

Overall, 88% (57 patients) completed 70% of the time points. Patients in the intervention arm were significantly more likely to complete 70% of time points than the control arm (97% vs 79%, *p* < 0.05).

**Fig. 3:**
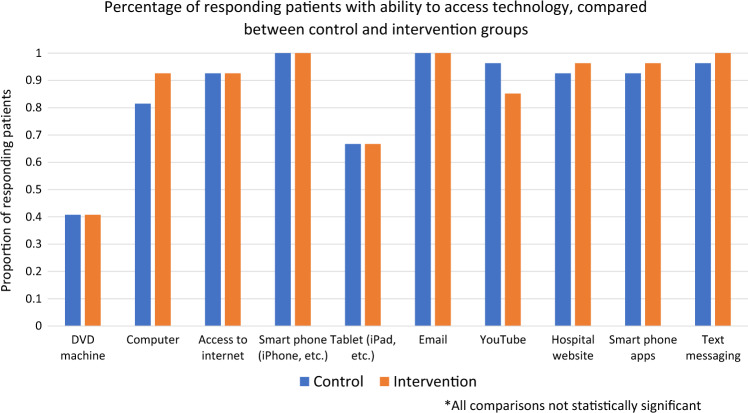
Percentage of responding patients with ability to access technology: a comparison between control and intervention groups

Of all 65 patients completing participation across both study arms, 54 completed the satisfaction tool (83.1%). Forty-three of those responding (80%) reported that telemonitoring was helpful to track their daily activities, and 51 (94%) stated that the timing of surveys was “just right.” The majority of patients (47 patients, 87%) did not feel that the survey questions were difficult to understand. Of note, all patients who consented to participate in this study had access to applicable technology at baseline, with 100% of responding patients reporting that they had access to a smart phone. Patient responses regarding access to technology are shown in Fig. 3.

## Discussion

This study is one of the first to incorporate PGHD in the form of vital signs, mobility, and ePROs, coupled with nursing intervention triggered by set thresholds [5], and examines a population that has the potential to derive significant benefit from remote telemonitoring after discharge [7], specifically patients undergoing complex gastrointestinal oncologic surgery. This analysis shows high levels of patient satisfaction and adherence with low rates of attrition in both study arms, suggesting that remote telemonitoring in this population is both feasible and acceptable to patients. Notably, higher levels of adherence were seen in the nursing supported intervention arm. Possible reasons for this finding include the receipt of personal reminders via telephone from nursing triage when the requested data points had not been entered in a timely fashion and patient knowledge that providers were anticipating the receipt of such data points as part of their overall postoperative care plan. Regardless, these results indicate that remote telemonitoring after complex gastrointestinal cancer operations appears both feasible and acceptable.

However, there are many questions to be answered regarding the applicability and utility of such an approach in the perioperative care of such patients. The patients who consented to participate in this study had a high level of baseline digital literacy, with 100% of participants indicating ownership of or access to a smartphone and the majority indicating that they had access to smartphone apps, internet, and text messaging. It remains unclear whether patients who did not have access to such technology at baseline would be comfortable consenting to a remote telemonitoring program such as the one offered here, as disparities in digital literacy have been associated with inability to participate in internet based health interventions [13]. Indeed, all three of the patients who initially consented to participate but later withdrew cited a reason of “feeling overwhelmed,” potentially due to the technological requirements of participation despite the assistance offered by study staff. In addition, patients who consented to participate in this study were relatively young, with a median age of 52 years. Although this may reflect the available patient population of the institution, the included patient population may therefore be biased toward those with a higher digital literacy at baseline, as older age has been previously associated with decreased comfort with health technology [14]. Thus, the roles of patient age, accessibility to applicable technology, and digital literacy in the successful use of remote telemonitoring after complex cancer operations will require further investigation.

This study is limited by several factors. Firstly, it was conducted at a single institution, which may limit its generalizability to the general population undergoing complex gastrointestinal oncologic operations, particularly given the young age and the high level of education achieved by this study’s participants. Furthermore, only those patients able to read and write in English were able to be included due to limitations of the technology used. Future studies will need to investigate the use of remote telemonitoring platforms for non-English speakers and those with limited literacy, as this may represent a source of disparity [15]. Additionally, as this manuscript entails feasibility, this study required a dedicated full time nurse to enroll and monitor patients. This can be a prohibitive cost in many settings. Finally, this study reflects a preliminary analysis in which only a portion of the patients ultimately consented to participate were included, and the metrics used to measure the endpoints of feasibility and accessibility may not fully capture the experience of remote telemonitoring in all patients. However, this interval analysis does indicate an adequate level of feasibility and accessibility, and further analysis will be able to be included in the final study results.

In conclusion, remote telemonitoring after discharge for patients undergoing complex gastrointestinal oncologic surgery appears feasible and acceptable, and higher levels of adherence are associated with the receipt of nursing triage outreach. Further research will be necessary to understand implications for the duration of telemonitoring, the impact on resource utilization and the potential to mitigate severity of complications by earlier identification.

### Supplementary Information

Below is the link to the electronic supplementary material.

Supplementary file1 (XML 88 KB)
